# Gene by environment interaction effects on the metabolic subtype of Polycystic Ovary Syndrome in Hispanic Community Health Study/Study of Latinos

**DOI:** 10.3389/fgene.2026.1801152

**Published:** 2026-08-28

**Authors:** Hridya R. Gardner, Michelle L. Meyer, Jennifer K. Wagner, Krista M. Perreira, Laura Y. Zhou, Martha Daviglus, Christina Cordero, Anne E. Justice, Lindsay Fernandez-Rhodes

**Affiliations:** 1 Department of Biobehavioral Health, Pennsylvania State University, University Park, PA, United States; 2 Department of Developmental Medicine, Geisinger Health System, Lewisburg, PA, United States; 3 Department of Population Health Sciences, Geisinger Health System, Danville, PA, United States; 4 Department of Emergency Medicine, University of North Carolina, Chapel Hill, NC, United States; 5 School of Engineering Design and Innovation, Pennsylvania State University, University Park, PA, United States; 6 Department of Social Medicine, University of North Carolina, Chapel Hill, NC, United States; 7 Department of Biostatistics and Health Data Science, Indiana University, Indianapolis, IN, United States; 8 Institute for Minority Health Research, University of Illinois at Chicago, Chicago, IL, United States; 9 Department of Psychology, University of Miami, Miami, FL, United States

**Keywords:** PCOS, PMOS, GxE, genetics, generalization, PRS, anxiety, Hispanic/Latinos

## Abstract

Polycystic Ovary Syndrome (PCOS) is a common polygenic endocrine disorder that is heterogenous in clinical presentation across genetic ancestry groups. PCOS is characterized by an array of symptoms such as hyperandrogenism, impaired mental health, and metabolic dysregulation. Studying the interaction of environmental factors (such as diet, physical activity, anxiety, and depression) with genetic variants on PCOS and its subtypes in populations with high cardiometabolic burden, e.g., Hispanic/Latinas, could aid in unraveling pathophysiological and genetic pathways through which PCOS functions. We sought to study gene by environment interactions with PCOS and its metabolic subtype (mPCOS) in a sample of US Hispanic/Latina female adults from the Hispanic Community Heath Study/Study of Latinos. In this large community-based study, we derived PCOS using self-reported condition and menstrual cycle information. We classified females with PCOS as having mPCOS if they had high metabolic impairment (fasting glucose, fasting insulin, or body mass index higher than the 75th percentile). There were 451 individuals with PCOS and 221 of them had mPCOS in our sample. We found that PCOS and mPCOS were significantly associated with hyperglycemia and high triglycerides in this population. While a polygenic risk score derived in European ancestry did not generalize to Hispanic/Latina females with PCOS, we identified the best proxy genetic variants in this population in known PCOS regions and investigated their interactions with four environment variables (diet, physical activity, anxiety and depression). Associations with known PCOS loci were generalized in our study at *STAG3L4* and *CACNA1G* genomic regions. We observed GxE interactions between variants in/near three genes and physical activity on PCOS and mPCOS, including *FGGY*, *FAT1*, and *PTHLH*. Additionally, we noted interactions between diet and a variant in *FANCC* on PCOS, and diet and a variant near *CMAS* on both PCOS and mPCOS. We also detected GxE interactions between anxiety and depression and a variant in *FGGY* on PCOS, and depression and a variant near *FBP1* on mPCOS. Our results point to potential protective effects of physical activity in females with PCOS and could inform future research on the mitigating effects of lifestyle management on PCOS genetic risk in Hispanic/Latino populations.

## 1 Introduction

The prevalence of Metabolic Syndrome (MetS) among United States (U.S.) Hispanic/Latino adults has increased from 32.9% in 2011 to 40% in 2021–2023 (see Box 1 for definitions and terms used) (Hirode and Wong, 2020; Abohashem et al., 2026). As a result, this population is disproportionately affected by higher rates of obesity, dyslipidemia, and Type 2 Diabetes Mellitus (T2D) (Hirode and Wong, 2020; Ford et al., 2002; Sam et al., 2015; Kazemi et al., 2022). Hispanic/Latina females also exhibit a higher prevalence of Polycystic Ovary Syndrome (PCOS) with rates as high as 18%–20% compared to a global prevalence of 6.5%–8% (Meyer et al., 2020; Mortada and Williams, 2015). With the increasing population size of Hispanic/Latino persons living in the US (50.5 million in 2010 to 62.1 million in 2020) (Parkinson and Schaeffer, 1996), they constitute an important ethnic group that requires clinical attention focused on improving cardiovascular disease (CVD) outcomes. The term Hispanic/Latino refers to a socially or culturally constructed identity self-reported by participants and it should not be construed as an indicator of genetic variation or genetic similarity to a particular ancestry. In fact, Hispanic/Latino individuals are known to be highly genetically diverse due to their widespread geographic origins, with reference populations from CLM (Colombian in Medellin, Colombia), MXL (Mexican Ancestry in Los Angeles, California, USA), PEL (Peruvian in Lima, Peru), and PUR (Puerto Rican in Puerto Rico), all being broadly classified under AMR or Admixed American superpopulation according to the 1000 Genomes Project (Conomos et al., 2016; Saccone et al., 2018; Setser et al., 2020; United States Cen sus Bureau, 2024). Hispanic/Latino populations often are also highly admixed with ancestries from African, European, and Amerindian populations (Manichaikul et al., 2012). Given the ancestral diversity within this population, studying the causal mechanisms can be challenging in cohort studies due to population stratification (Conomos et al., 2016), sociocultural factors, and linkage disequilibrium (LD) patterns (Sharma et al., 2025). Studies that analyze the genetic risk of complex traits in Hispanic/Latinos, such as PCOS, are largely lacking in the literature (Mills and Rahal, 2020).

BOX 1 **Terms and definitions.**

According to the definition set forth by U.S. Office of Management and Budget that is also adopted by U.S. Census and the Hispanic Community Health Study/Study of Latinos (HCHS/SOL), Hispanic/Latino refers to a person of Cuban, Mexican, Puerto Rican, South or Central American, or of other Spanish culture or origin, regardless of race (United States Cen sus Bureau, 2024). Groups like Dominicans may also be included in Hispanic/Latino individuals (Conomos et al., 2016). Females from this ethnic group are referred to as Hispanic/Latina to align with the conventions of the Spanish language.

PCOS is an endocrine disorder associated with multiple adverse health outcomes, including subfertility (Joham et al., 2016), MetS (Mortada and Williams, 2015; Lee et al., 2022; Moran and Norman, 2004), insulin resistance (Dunaif et al., 1996), CVD (Dokras, 2008), hypertension (Kim et al., 2023; Giallauria et al., 2008), mental health disorders (Hollinrake et al., 2007; Chaudhari et al., 2018; Yin et al., 2021), poor body image (Deeks et al., 2011), endometrial cancer (Daniilidis and Dinas, 2009; Hardiman et al., 2003), and other comorbidities. It affects 1 in 10 women in the U.S. (Azziz, 2006; Barthelmess and Naz, 2014) and twice as many Hispanic/Latinas (Goodarzi et al., 2005; Zhao and Qiao, 2013). PCOS generally manifests during puberty, and women who are predisposed to PCOS demonstrate many endocrine-related characteristics at this stage such as hyperactive androgen secretion, low levels of insulin-like growth factors binding protein-1 (IGFBP-1), and lower sex hormone binding globulin (SHBG) (Nobels et al., 1992). These characteristics persist through adulthood and interfere with other physiologic processes in the body. Several biological processes related to inflammation in the adipocytes and secretion of adipokines demonstrate the foundational importance of insulin sensitivity in the development of PCOS with affected individuals often presenting with underlying insulin resistance (Diamanti-Kandarakis and Dunaif, 2012; Rojas et al., 2014/01). Fatty acids from the central adipose tissues enter the bloodstream, supplying more substrate to the liver for triglyceride production, resulting in elevated triglycerides in women with PCOS (Farrell and Antoni, 2010; Moran et al., 2015). This association has been previously validated in the Hispanic Community Health Study/Study of Latinos (HCHS/SOL) participants by our group and other HCHS/SOL investigators, also linking PCOS to MetS, inflammation, impaired fasting glucose (FG), and high triglycerides (Meyer et al., 2020; Rao et al., 2024).

Clusters within PCOS have been delineated and studied based on key PCOS hormones and genes (Dapas et al., 2020; Chen et al., 2024; Stamou et al., 2024). The metabolic subtype of PCOS (mPCOS), which is characterized by higher fasting insulin (FI), higher FG, higher body mass index (BMI), lower luteinizing hormone (LH), and lower follicular stimulating hormone (FSH), was derived by Dapas et al. They leveraged a genome-wide association study (GWAS) by Hayes and colleagues (Hayes et al., 2015) and identified a strong association between mPCOS and the *KCNH7/FIGN* locus in European ancestry (Dapas et al., 2020). We aimed to derive mPCOS in Hispanic/Latinas and generalize its association with several known PCOS loci, identified in a European ancestry by Hayes et al., to this population (Hayes et al., 2015).

GWAS of PCOS have identified several key variants in genes such as *THADA, INSR, DENND1A, FSHB, ARL14EP* (Hayes et al., 2015; Day et al., 2018; Tian et al., 2020; Li et al., 2012; Chen et al., 2011). We also used summary statistics from a large GWAS of PCOS conducted by Day and colleagues, conducted in a European ancestry sample, to test the transferability of a polygenic risk score (PRS) to Hispanic/Latinas (Day et al., 2018). Given the higher MetS burden in this population, analyzing the predictive power of this PRS in Hispanic/Latinas in HCHS/SOL will be a pivotal step in precision medicine as most PRS studies are conducted in primarily European ancestry samples.

Testing the association of the PCOS PRS with mPCOS and studying its interactions with environment factors could improve the understanding of the combined risk imparted due to PCOS genetic predisposition and the environment. Examining the interactions with cardiometabolic factors such as FG, higher BMI, and high triglycerides will further help in dissecting the gene-environment interactions (GxE) and reveal the moderating effect of these clinical features on the relationship between single nucleotide polymorphisms (SNP) and PCOS. To our knowledge, there have been limited studies on PCOS PRSs (Zhang B. et al., 2020; Actkins et al., 2023; Joo et al., 2020), while studies analyzing the effect of GxE interactions on mPCOS are non-existent in the PCOS literature, to date.

Potential environmental factors related to PCOS pathophysiology include diet, physical activity, anxiety, and depression (Brutocao et al., 2018; Hambleton et al., 2022; Merkin et al., 2016). Recent reviews of the extant PCOS literature on lifestyle interventions noted that a balanced and nutrient-rich diet (consisting of 40% energy from carbohydrates, 30% from fats, and 30% from protein, along with high-fiber foods, omega-3 fatty acids, antioxidants and anti-inflammatory foods) has been recommended for PCOS patients (Shahid et al., 2022; Gautam et al., 2025). The prevalence of diagnosed anxiety disorders in women with PCOS is roughly 30%, with comorbid depressive disorders affecting between 11%–25% of this population (Chaudhari et al., 2018).

Individuals with PCOS tend to have negative body image, low self-esteem and generally lower quality of life compounded by metabolic disturbances later in life (Deeks et al., 2011; Teede et al., 2010). Personalized interventions addressing both physical and mental challenges in Hispanic/Latinas with PCOS could benefit from the identification of gene targets relevant in this population that is disproportionately burdened by metabolic disease and aid in advancing translational efforts. Moreover, GxE interactions with psychological features are understudied and in Hispanic/Latinas, analyzing these models could unlock a path forward in mitigating PCOS from metabolic, lifestyle, and psychological perspectives. To this end, this study aims to 1) evaluate the generalizability of associations with known PCOS genetic loci to Hispanic/Latina females from HCHS/SOL, 2) assess the transferability of a European-ancestry-based PRS, and 3) analyze the interactions of variants and the PRS with several environmental factors.

## 2 Methods

### 2.1 Study information

The HCHS/SOL was designed to study the prevalence and incidence of chronic diseases (e.g., diabetes, CVD, and pulmonary diseases) and understand the risk and protective factors for morbidity and mortality in this population (Sorlie et al., 2010). A sample of 16,415 self-identified Hispanic/Latino men and women between the ages of 18–74 years was recruited from 2008 to 2011 from four communities across the US–Bronx, NY; Chicago, IL; Miami, FL; and San Diego, CA–in a two-stage probability-based sampling method to ensure diversity in recruitment (LaVange et al., 2010). The HCHS/SOL cohort has been followed up annually after Visit 1 (V1) for identification of hospitalizations and in the clinic as part of a Visit 2 (11,623 participants returning for V2; 2014–2017) and subsequent visits, with assessments including both cardiovascular and reproductive/pregnancy complications. HCHS/SOL collected responses to questionnaires across several domains in both English and Spanish. At V2 specifically, participants’ reproductive medical history was assessed which included their self-report of a previous diagnosis of PCOS. At V1, participants were asked to self-report their Hispanic/Latino background (or heritage) with the question “Which of the following best describes your Hispanic/Latino heritage?”, and the following options: Dominican or Dominican descent, Central American or Central American descent, Cuban or Cuban descent, Mexican or Mexican descent, Puerto Rican or Puerto Rican descent, South American or South American descent, more than one heritage, or other. Then the study personnel categorized them as belonging to Dominican, Central American, Cuban, Mexican, Puerto Rican, South American, or of mixed heritage. The study was approved by the institutional review boards at all participating institutions and informed consent was obtained from all participants.

### 2.2 Subjects

All females from V2 were included in the current study and PCOS was characterized as having either the signs of PCOS, such as irregular menstrual cycles (cycles that last longer than 35 days, or reported as “too irregular to say”), or having answered “yes” to a self-report question on PCOS. In addition, we restricted our sample to pre-menopausal females and those of reproductive age. Postmenopausal status was defined by the presence of at least one year of amenorrhea, whether natural, surgical (hysterectomy, oophorectomy, or ablation), or induced by medical therapy (chemotherapy, radiation, or hormonal suppression). The final sample was also restricted to females aged 18–55 (based on their V1 age and their follow-up time) (Zhu et al., 2019).

**FIGURE 1:**
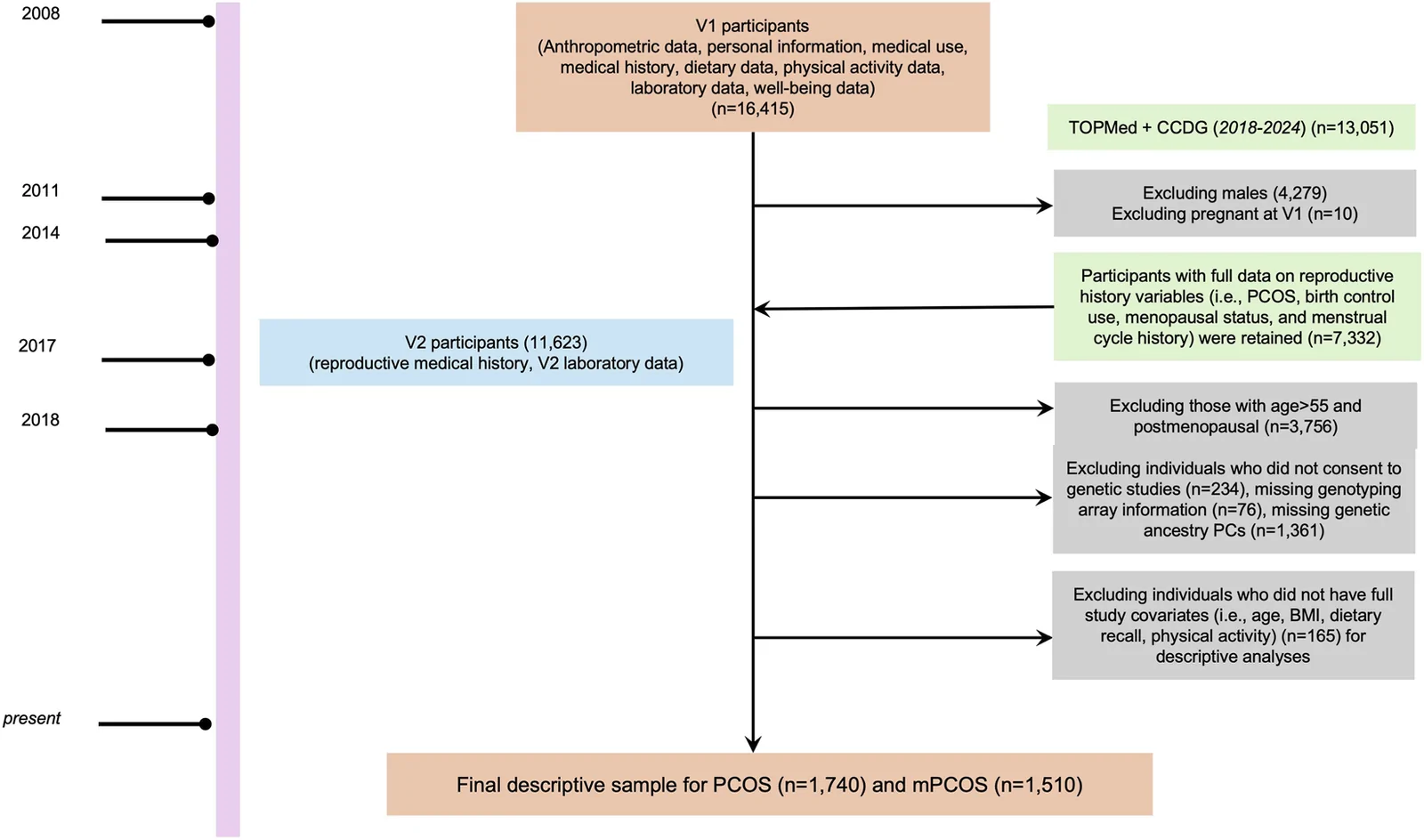
Study inclusions and exclusions. Figure shows the HCHS/SOL study timeline, inclusion and exclusion criteria and final sample sizes. Abbreviations: TOPMed: Trans-Omics for Precision Medicine, CCDG: Common disease genomics consortium, mPCOS: metabolic subtype of PCOS, PC: principal components.

The final sample consisted of 451 females with PCOS and 1,289 without PCOS (Figure 1). A subset of individuals with PCOS were characterized as having mPCOS if they had BMI, FG, or FI greater than the 75^th^ percentile (n = 221). Annual household income, education level, marital status, health insurance coverage, US nativity, and several other demographic characteristics were also collected. We excluded those with missing data on reproductive medical history or genetic ancestry principal components (PCs). We also excluded those with missing dietary recall or incomplete physical activity data. Participants who consented for the genetic analyses (Gonzalez et al., 2021), those who were premenopausal and of reproductive age at V2, and those with complete genotype information were retained as shown in Figure 1.

### 2.3 Genotyping and imputation in HCHS/SOL

HCHS/SOL participants were genotyped in multiple freezes differing based on an individual’s consent for genetic data sharing, with 11,389 (out of the 11,623 returning for V2) consenting to the use of their DNA samples for genetic studies conducted by HCHS/SOL - affiliated investigators and having reproductive medical history and laboratory data (Kowalski et al., 2019). Efforts included genotyping on both the Illumina Omni2.5M array (HumanOmni2.5-8 version 1-1 with 2,293,715 SNPs including ancestry-informative markers from Amerindian population) and the Illumina Multi-Ethnic Genotyping Array (MEGA) array (consisting of 1,705,969 SNPs), the latter being a part of the Population Architecture using Genomics and Epidemiology (PAGE) consortium to improve the assessment of genetic variation in non-European populations (Kowalski et al., 2019). Genotyped variants underwent quality control to apply technical filters, remove variants with missing call rate, remove duplicated variants and variants with Mendelian errors, remove variants with Hardy-Weinberg *P* < $1\times 10^{-4}$ , and other filters described in detail in previous publications (Chang et al., 2015; Wojcik et al., 2019). Due to its focus on non-European genetic variants, we leverage the second genotyping effort (Kowalski et al., 2019) and HCHS/SOL investigators have carried 1,402,653 MEGA variants remaining post-quality control forward to imputation on the National Heart Lung and Blood Institute’s Trans-Omics in Precision Medicine (TOPMed) panel and imputation server (Kowalski et al., 2019; Rao et al., 2025) (Figure 1).

### 2.4 Anthropometric measurements

Standing height was measured with a fixed stadiometer and a vertical backboard when participants were barefoot during their anthropometric assessment (Kaplan et al., 2014). Study personnel asked for self-report of weight (kg or lb) before measuring the participant’s body weight (kg). Waist circumference in centimeters was measured at the level of the upper border of the iliac crest, and this was done using Gulick II 150 and 250 tapes (Sammons Preston, Chicago, IL, USA), while the hip measurement was taken at the maximum circumference of the buttocks. Waist and hip measurements in centimeters were divided to obtain the waist to hip ratio and BMI was calculated by using body weight in kilograms and the average of two height measurements in meters squared.

### 2.5 Cardiometabolic health factors

During V1 and V2 of HCHS/SOL, participant information on laboratory measures such as FG, FI, blood pressure, high-density lipoprotein (HDL), low-density lipoprotein (LDL), triglycerides, homeostatic model of assessment for insulin resistance (HOMA-IR), lipids, and other measures were also collected. Participants were asked to fast for 12 h before venous blood draws, except in cases where this was not possible due to the participant’s medical condition. Blood draws were performed by trained phlebotomists, and appropriate biospecimen storage and quality control procedures were carried out (Diaz et al., 2017). FG, FI, and lipids were derived from blood, and the average of three separate systolic and diastolic blood pressures in millimeters of mercury (mmHg) were obtained using standard procedures (Diaz et al., 2017).

### 2.6 Environmental factors

Dietary interviews were conducted and processed at the Nutrition Reading Center. Interviews consisted of two non-consecutive 24-h recalls, the first one administered at V1 in-person and the second one, by phone within a month for the first recall (Isasi et al., 2014). The nutrition data system for research (NDS-R) software was used to classify food groups reported by the participants based on counts and components. HCHS/SOL study personnel sorted the reported ingredients and recipes into food groups which were summed and averaged across visits for each participant. The final measure of the Alternative Healthy Eating Index, AHEI2010, which was composed of 11 dietary components, was constructed by scoring each food group, with higher scores indicating a healthier diet. Scoring criteria for AHEI2010 have been described previously (Chiuve et al., 2012).

Physical activity interviews were conducted by trained interviewers using the World Health Organization’s Global Physical Activity Questionnaire (GPAQ) (Armstrong and Bull, 2006). This 16-item questionnaire investigated activity at work, during transportation, and during leisure time, and assessed overall sedentary behavior during a “typical week”. Total activity was summed in Metabolic Equivalents per day (MET-minute/day), and this measure was used in the present study. GPAQ is a well-validated questionnaire used across countries and is one of the most frequently used measures for physical activity level ascertainment (Bull et al., 2009; Keating et al., 2019).

### 2.7 Psychological measures

Participants were asked at V1 to indicate their general tendency to experience anxiety symptoms on the Trait version of the Spielberger State Trait Anxiety Inventory 10-item scale, with items ranked from 0 (almost never) to 3 (almost always). Total scores were summed and ranged from 10 to 40, with higher scores indicating more anxiety (Camacho et al., 2015).

Depression symptoms were measured using the Center for Epidemiological Studies Depression-10 item scale (CESD-10) which has been shown to have strong psychometric properties overall in Hispanic/Latino populations (Smarr and Keefer, 2011; González et al., 2019). Participants were asked to indicate how often they had experienced depression symptoms in the 7 days, with each item ranked from 0 (rarely or none of the time) to 3 (most or all of the time), yielding a total score ranging from 0 to 30.

### 2.8 Cardiometabolic outcomes

Diabetes status at V2 was derived using a combination of antidiabetic medication use in the 4 weeks prior to V2, self-report of diabetes, and a lab report of diabetes quantified by FG greater than 126 mg/dL based on the American Diabetes Association guidelines (American Diabetes Association, 2025).

Metabolic Syndrome was characterized if the participant had three of the following five criteria as per the National Cholesterol Education Program Third Adult Treatment Panel (NCEP/ATP III) criteria – (Hirode and Wong, 2020) elevated blood pressure (systolic >130 mmHg and diastolic >85 mmHg) or taking antihypertensive medication (Abohashem et al., 2026), had triglyceride concentration greater than 150 mg/dL (Ford et al., 2002), had HDL concentration lesser than 50 mg/dL (Sam et al., 2015), had FG greater than 100 mg/dL or reported the use of anti-diabetic medication, or (Kazemi et al., 2022) had high waist circumference (88> cm) (Cleeman, 2001).

### 2.9 Statistical analyses

We performed analyses to provide descriptive statistics of study covariates such as age, self-report of Hispanic/Latino background, income, education, cardiometabolic outcomes, reproductive outcomes, and four environmental factors (diet, physical activity, anxiety and depression). We also tested for differences in them by PCOS and mPCOS categories using Wilcoxon-rank sum tests for continuous variables and Pearson chi-squared tests for categorical variables.

Associations of cardiometabolic and environmental factors with PCOS and mPCOS statuses were tested in generalized linear models adjusting for age, self-reported Hispanic/Latino background, and study center. HCHS/SOL oversampled at both stages of the probability sampling to increase households with adults aged 45–74 years and this resulted in participants selected with unequal probabilities in the final cohort. To account for this over-sampling and differential non-response at the household and person level, HCHS/SOL employed the use of complex survey sampling weights. These weights also serve to trim extreme values and adjust the weights to known population distributions. Therefore, our regression models were implemented in R *surveyglm* function accounting for HCHS/SOL complex sampling weights at V2, clusters, and primary sampling units.

#### 2.9.1 Incorporating a PCOS polygenic risk score

Summary statistics from a GWAS of PCOS conducted in European ancestry was used to derive a PRS in PRS-CSx software (Day et al., 2018; Ruan et al., 2022). PRS-CSx is a python-based tool that compiles GWAS results with LD information from reference panels across various populations to infer cross-population weights for SNPs. LD reference files for each of the 1,000 Genomes global population groups–AFR, EUR, AMR, EAS, or SAS–and their SNP information file were used to calculate the posterior effect sizes for individual SNPs. PRS-CSx analysis was conducted in unrelated females identified using the KING-robust kinship estimator in PLINK 2.0 (Manichaikul et al., 2010; Chang and Vattikuti, 2025). In instances of relatedness in the female HCHS/SOL sample (third degree or closer), we applied a priority-based pruning approach, ensuring that participants meeting both PCOS and mPCOS diagnostic criteria were retained over single-status cases or controls. Posterior SNP effect size estimates were utilized to calculate a summed score across all variants for each individual in PLINK (Chang et al., 2015). To assess the predictive performance of the PRS and to plot the area under covariate-adjusted Receiver Operating Characteristic curve (AROC-AUC), we used the R package *ROCnReg* (Rodriguez-Alvarez and Inacio, 2021). The weighted score sum was used in association analyses with PCOS and mPCOS and was adjusted by age and first five genetic ancestry principal components. We tested for modified genetic susceptibility by fitting logistic regression models that incorporated a cross-product term between the PRS and each environmental factor, treating both PCOS and mPCOS as the dependent variables.

#### 2.9.2 Generalization of known PCOS associations and gene by environmental interactions

We selected SNPs with p-value less than 1 × 10^−6^, filtered from a previous GWAS of PCOS by Hayes and colleagues (Hayes et al., 2015) and tested their association with PCOS and mPCOS in the sample. A total of 329 out of 340 variants remained in the HCHS/SOL sample after quality control (Supplementary Table S1). For each GWAS significant variant reported in the Hayes publication, we also identified the best genetic proxy in our study within 500 kb of the top signal from Hayes study. GxE interactions were estimated for each environmental factor (diet, physical activity, anxiety, and depression, all modeled continuously) with variants in ±500 kb region around the 329 GWAS significant SNPs from Hayes publication that were present in our study. Our genetic analyses modeled SNP genotype dosages continuously (0–2 minor alleles) under an additive genetic model. We used SUGEN v8.9 (Lin et al., 2014) for genetic analyses, which is an analytic tool developed for the analysis of HCHS/SOL data that implements a generalized estimating equation to account for relatedness and can account for its complex sampling weights. We used cluster IDs derived from our kinship output to adjust for genetic relatedness. Regression analyses were run in single variant models and adjusted for age at V2 and first five genetic ancestry PCs. For each GxE model, we estimated p-values for the main genetic effect, the environmental effect, and the interaction term. Additionally, a joint 2-df test was performed to capture variants with significant combined effects. To maintain a stringent false discovery rate, the Bonferroni threshold was set at p < 0.0004, accounting for 31 independent tests across four environmental factors (and suggestive significance was set at a p-value threshold of 0.05).

## 3 Results

We identified 451 pre-menopausal participants with PCOS (221 with mPCOS) of reproductive age (18 ≤ age ≤ 55 years), with a median age of 39 years (Table 1). A test for differences in age between females with PCOS and without PCOS indicated that there was a significant difference (Wilcoxon rank sum p < 0.05), but not between mPCOS and the control group (Table 1). Overall, roughly half of the reproductive aged females in the communities studied in HCHC/SOL were of Mexican heritage, 12% were of Central American background, 11% Puerto Rican, 11% Dominican, 8.6% Cuban, 6.7% South American, and 3.7% with more than one heritage or other. The study sample was approximately evenly distributed between the two income categories (household income less than $30,000 per year and $30,000 or more per year). Sixty one percent of the sample reported educational attainments and training beyond high school. There were no significant differences in education levels by PCOS or mPCOS status based on Pearson chi-squared test (p > 0.05). Thirty four percent of the participants reported having health insurance coverage and there were significantly more insured individuals in the PCOS “no” status compared to PCOS “yes” status (p < 0.05). The distribution of insured individuals across mPCOS categories did not significantly differ. Roughly 70% of the sample reported being US native born (i.e., born in the US or its territories, including Puerto Rico) – 61% of those with PCOS and 58% of those with mPCOS–and these statuses significantly differed by US nativity (both p < 0.001). Roughly half of the sample was married or living with a partner, 36% reported being single, and 11% reported being separated, divorced, or widowed.

**TABLE 1:** Descriptive statistics of demographic characteristics in HCHS/SOL females stratified by PCOS and mPCOS categories.

Characteristic	N = 1,289	Overall (n = 1,740) N, (%)*	Control (n = 1,289) N, (%)*	PCOS (n = 451)		mPCOS (n = 221)	
				Yes	p-value	Yes	p-value
AGE at V2	1,740	41 (32, 47)	41 (33, 47)	39 (30, 46)	**<0.001**	39 (31, 46)	**0.07**
Self report of Hispanic/Latino background^	1,737	​	​	​	**<0.001**	​	**<0.001**
Dominican	​	191 (11%)	151 (12%)	40 (8.9%)	​	18 (8.1%)	​
Central american	​	216 (12%)	168 (13%)	48 (11%)	​	168 (13%)	​
Cuban	​	149 (8.6%)	124 (9.6%)	25 (5.6%)	​	124 (9.6%)	​
Mexican	​	805 (46%)	589 (46%)	216 (48%)	​	589 (46%)	​
Puerto rican	​	196 (11%)	134 (11%)	62 (14%)	​	134 (10%)	​
South american	​	116 (6.7%)	86 (6.7%)	30 (6.7%)	​	86 (6.7%)	​
More than one heritage/Other	​	64 (3.7%)	35 (2.7%)	29 (6.4%)	​	35 (2.7%)	​
DK/Refused	​	0	0	0	​	0	​
Income level	1,592	​	​	​	0.30	​	0.50
Less than $30,000	​	874 (55%)	654 (56%)	220 (53%)	​	111 (53%)	​
$30,000 or more	​	718 (45%)	522 (44%)	196 (47%)	​	98 (47%)	​
Education level	1,740	​	​	​	0.08	​	0.20
Less than high school	​	374 (16%)	218 (17%)	56 (12%)	​	26 (12%)	​
High school or equivalent	​	405 (23%)	297 (23%)	108 (24%)	​	52 (24%)	​
Greater than high school or equivalent	​	1,061 (61%)	774 (60%)	287 (64%)	​	143 (65%)	​
Has insurance coverage	1,631	​	​	​	**0.03**	​	0.20
Yes	​	547 (34%)	423 (35%)	124 (29%)	​	64 (30%)	​
No	​	1,084 (66%)	784 (65%)	300 (71%)	​	147 (70%)	​
Born in US 50 states or Puerto Rico	1,737	​	​	​	**<0.001**	​	**<0.001**
Yes	​	1,235 (71%)	960 (75%)	275 (61%)	​	129 (58%)	​
No	​	503 (29%)	327 (25%)	175 (39%)	​	92 (42%)	​
Marital status	1,735	​	​	​	0.20	​	0.12
Single	​	630 (36%)	452 (35%)	178 (40%)	​	89 (40%)	​
Married or living with a partner	​	910 (52%)	684 (53%)	226 (50%)	​	114 (52%)	​
Separated, divorced, or widowed	​	195 (11%)	150 (12%)	45 (10%)	​	17 (7.7%)	​

Descriptive statistics of demographic characteristics in HCHS/SOL females are presented, stratified by PCOS status and mPCOS subtype (each compared against a shared control group). *Continuous variables are reported as median (Q1, Q3), with p-values derived from the Wilcoxon rank-sum test. Categorical variables are reported as percentages, with p-values derived from Pearson’s chi-square tests evaluating the association between each characteristic and case status (PCOS vs. Control and mPCOS vs. Control). A two-sided p-value of <0.05 was used to define statistical significance. All significant differences were bolded. Abbreviations: mPCOS, metabolic subtype of PCOS; DK, Don’t know.

^Measure only available at V1.

BMI, waist-to-hip ratio, FG, FI, triglycerides, HDL cholesterol and HOMA-IR were significantly higher in individuals with mPCOS compared to those without (p < 0.05) (Table 2). The median BMI in individuals with PCOS was 30 kg/m^2^ and in individuals with mPCOS was 35 kg/m^2^, however the difference in BMI by PCOS status was not significant. HOMA-IR and FI were significantly higher in those with PCOS compared to those without (p < 0.05). Median HOMA-IR was 2.99 in those with PCOS and 5.45 in those with mPCOS compared to 2.74 in controls. Diabetes status differed significantly by PCOS and mPCOS groups (p < 0.01) but not metformin use. The presence of PCOS or mPCOS was strongly associated with cycle length (p < 0.001), with over half of affected individuals reporting irregular cycles. Significantly more individuals with PCOS and mPCOS reported having infertility at V2 compared to the control group (22% in those with PCOS and 24% in those with mPCOS vs. 10% in controls). Anxiety symptom scores varied significantly by outcome group (p < 0.05), where the median score was 17 in those with PCOS and 18 in those with mPCOS. CESD-10 scores and total physical activity did not differ significantly between individuals with and without the outcomes. Individuals with PCOS also reported significantly lower healthy eating index compared to those without PCOS (p < 0.05) but the index did not differ significantly by mPCOS status. Overall, it was observed that there were worse metabolic and reproductive outcomes in those with PCOS compared to those without PCOS, and for certain outcomes, these measures were more impaired in those with mPCOS.

**TABLE 2:** Descriptive statistics of laboratory measures and health outcomes stratified by PCOS and mPCOS.

Characteristic	N	Overall	Controls	PCOS		mPCOS	
		N = 1,740 N(%)*	N = 1,289 N(%)*	Yes	p-value	Yes	p-value
				N = 451 N(%)*		N = 221 N(%)*	
Body Mass index at V2 (kg/m^2^)	1,661	29 (25, 34)	29 (25, 34)	30 (26, 35)	0.14	35 (30, 40)	**<0.001**
Waist to hip ratio at V2	1,656	0.89 (0.84, 0.94)	0.89 (0.84, 0.94)	0.90 (0.85, 0.94)	**0.002**	0.92 (0.87, 0.98)	**<0.001**
Fasting glucose (mg/dL)	1,726	93 (88, 100)	93 (88, 100)	93 (88, 100)	0.70	100 (93, 108)	**<0.001**
Triglycerides (mg/dL)	1,726	87 (60, 125)	87 (60, 124)	88 (62, 127)	0.30	109 (78, 158)	**<0.001**
HDL-cholesterol (mg/dL)	1,727	51 (43, 61)	51 (43, 61)	50 (41, 60)	0.20	46 (39, 52)	**<0.001**
LDL-cholesterol (mg/dL)	1,704	106 (88, 127)	107 (88, 127)	104 (86, 128)	0.40	108 (88, 132)	0.50
Fasting insulin (mU/L)	1,725	12 (8, 18)	12 (8, 17)	13 (8, 21)	**0.003**	21 (16, 28)	**<0.001**
HOMA index of insulin resistance	1,725	2.78 (1.77, 4.46)	2.74 (1.77, 4.21)	2.99 (1.80, 5.43)	**0.01**	5.45 (3.92, 7.94)	**<0.001**
Diabetes by self-report, lab measure, or medication use	1,740	​	​	​	**0.004**	​	**<0.001**
No	​	1,464 (84%)	1,104 (86%)	360 (80%)	​	147 (67%)	​
Yes	​	276 (16%)	185 (14%)	91 (20%)	​	74 (33%)	​
Metformin use at V1 or V2	1,728	​	​	​	0.8	​	0.05
No	​	1,681 (97%)	1,248 (97%)	433 (97%)	​	206 (95%)	​
Yes	​	47 (2.7%)	34 (2.7%)	13 (2.9%)	​	11 (5.1%)	​
Cycle length	1,736	​	​	​	**<0.001**	​	**<0.001**
Less than 24 days	​	119 (6.9%)	100 (7.8%)	19 (4.2%)	​	9 (4.1%)	​
25–35 days	​	1,322 (76%)	1,170 (91%)	152 (34%)	​	71 (32%)	​
More than 35 days	​	48 (2.8%)	0 (0%)	48 (11%)	​	24 (11%)	​
Too variable or irregular	​	230 (13%)	0 (0%)	230 (51%)	​	116 (53%)	​
Refused	​	0 (0%)	0 (0%)	0 (0%)	​	0 (0%)	​
Unknown	​	17 (1.0%)	16 (1.2%)	1 (0.2%)	​	0 (0%)	​
Birth control used ever	1,737	​	​	​	**0.01**	​	0.05
No	​	521 (30%)	409 (32%)	112 (25%)	​	56 (25%)	​
Yes	​	1,216 (70%)	877 (68%)	339 (75%)	​	165 (75%)	​
Birth control use at V2	1,740	​	​	​	**<0.001**	​	**<0.001**
No	​	1,649 (95%)	1,239 (96%)	410 (91%)	​	201 (91%)	​
Yes	​	91 (5.2%)	50 (3.9%)	41 (9.1%)	​	20 (9.0%)	​
Infertility for >1year	1,735	​	​	​	**<0.001**	​	**<0.001**
No	​	1,508 (87%)	1,156 (90%)	352 (78%)	​	168 (76%)	​
Yes	​	226 (13%)	129 (10%)	97 (22%)	​	53 (24%)	​
Unknown	​	1 (<0.1%)	1 (<0.1%)	0 (0%)	​	0 (0%)	​
Smoking at V2	1,739	​	​	​	0.60	​	0.7
No	​	1,541 (89%)	1,144 (89%)	397 (88%)	​	198 (90%)	​
Yes	​	198 (11%)	144 (11%)	54 (12%)	​	23 (10%)	​
10-Item state-trait anxiety inventory summary score^a^	1,711	17.0 (13.0, 22.0)	17.0 (13.0, 21.0)	17.0 (13.5, 22.0)	**0.03**	18.0 (14.0, 23.0)	**0.017**
10-Item center for epi depression summary score^a^	1,715	6 (3, 11)	6 (3, 10)	6 (3, 11)	0.20	7 (3, 11)	0.30
Alternative healthy eating index 2010^a^	1,726	46 (41, 51)	46 (41, 51)	45 (40, 50)	**0.01**	46 (41, 51)	0.70
Total physical activity (MET-min/day)^a^	1,730	171 (29, 583)	161 (26, 577)	171 (40, 583)	0.40	191 (37, 664)	0.30

Desciriptive summary statistics of lab measures and clinical health outcomes have been represented here stratified by PCOS status and mPCOS subtype (each compared against a shared control group). *Continuous variables are reported as median (Q1, Q3), with p-values derived from the Wilcoxon rank-sum test. Categorical variables are reported as percentages, with p-values derived from Pearson’s chi-square tests evaluating the association between each characteristic and case status (PCOS vs. Control and mPCOS vs. Control). A two-sided p-value of <0.05 was used to define statistical significance. All significant differences were bolded.

^a^ Measure only available at V1.

### 3.1 Regression analyses of cardiometabolic and environmental factors by PCOS and mPCOS status

Regressing cardiometabolic and environmental factors on PCOS and mPCOS status revealed several notable associations (Table 3). Individuals with PCOS exhibited significantly higher waist-to-hip ratio, FI, HOMA-IR, and triglycerides compared to those without the condition even after adjusting for age, Hispanic/Latino background, and study center (all *β* > 0, p < 0.01). Similar trend for these factors was observed with mPCOS and additionally, BMI, diastolic blood pressure, FG, HDL, and LDL were also significantly elevated in those with mPCOS (all *β* > 0, p < 0.0001). There were no significant associations noted between the four environmental factors (diet, physical activity, anxiety symptoms and depression symptoms) and PCOS or mPCOS statuses in our regression analyses.

**TABLE 3:** Association of cardiometabolic and environmental factors with PCOS and mPCOS in HCHC/SOL.

​	PCOS “Yes” (n = 451) vs. “No” (n = 1,289)			mPCOS “Yes” (n = 221) vs. “No” (n = 1,289)		
Cardiometabolic and environmental factors	N	*β*(SE)	p-value	N	*β*(SE)	p-value
Body Mass index (kg/m^2^)	1,658	0.54 (0.49)	0.42	1,441	5.18 (0.7)	**<0.0001**
Waist to hip ratio at V2	1,653	**0.02 (0.01)**	**0.0003**	1,436	0.04 (0.01)	**<0.0001**
SBP, mmHg	1736	−0.04 (0.85)	0.96	1,507	0.85 (1.19)	0.48
DBP, mmHg	1735	0.61 (0.71)	0.39	1,506	2.26 (0.94)	**0.02**
Fasting glucose, mg/dL	1723	1.26 (2.23)	0.57	1,497	12.58 (4.01)	**0.002**
Fasting insulin, mg/dL	1722	**2.53 (0.83)**	**0.003**	1,496	10.75 (1.20)	**<0.0001**
HOMA-IR	1722	**0.80 (0.25)**	**0.002**	1,496	3.32 (0.41)	**<0.0001**
Triglycerides, mg/dL	1723	**14.78 (5.34)**	**0.006**	1,497	0.04 (0.01)	**<0.0001**
HDL, mg/dL	1724	−0.27 (0.99)	0.79	1,498	−6.67 (0.87)	**<0.0001**
LDL, mg/dL	1701	1.45 (2.08)	0.49	1,476	7.75 (2.51)	**0.002**
Diet quality (AHEI-2010)	1725	−0.46 (0.36)	0.20	1,497	−0.29 (0.58)	0.64
Total physical activity (MET-min/day)	1729	65.71 (61.39)	0.28	1,501	0.85 (1.19)	0.48
Anxiety symptoms (STAI)	1711	0.59 (0.42)	0.16	1,491	0.75 (0.53)	0.16
Depression symptoms (CESD10)	1715	0.49 (0.40)	0.23	1,494	0.36 (0.49)	0.46

Data are presented as adjusted *β* coefficients, their corresponding standard errors (SE), and model sample sizes (N). Models were conducted using survey-weighted generalized linear regression to estimate the association between PCOS and mPCOS statuses (Reference: “No”) and cardiometabolic and behavioral outcomes. mPCOS represents a subset of individuals categorized as having PCOS with additional metabolic complications within the HCHS/SOL cohort.

Abbreviations: AHEI 2010, American Healthy Eating Index 2010; BMI, body mass index; CESD10; Center for Epidemiologic Studies Depression Scale; CI, Confidence Interval; DBP, diastolic blood pressure; HDL, high density lipoprotein; HOMA-IR, Homeostatic model assessment of Insulin Resistance; LDL, low density lipoprotein; OR, Odds ratio; SBP, systolic blood pressure; SD, standard deviation; STAI, State-Trait Anxiety Index

All models were adusted for HCHS/SOL complex sampling procedures and implemented in R *surveyglm()*. All models were adjusted for age at V2, self-reported Hispanic/Latino background, and study center.

### 3.2 Polygenic risk score analyses

We tested the transferability of a PCOS PRS derived from summary statistics of GWAS and meta-analysis conducted in European-ancestry population (Day et al., 2018) to reproductive aged females in HCHC/SOL using PRS-CSx (Ruan et al., 2020). The European-ancestry PRS, computed using genotype dosages across roughly a million SNPs, did not successfully predict PCOS or mPCOS outcomes in this study (AROC-AUC<0.8) (Supplementary Figures S1A,B). As expected, in the regression analyses testing the interactions between the PCOS PRS and study characteristics on PCOS and mPCOS, no significant PRSxE effects were detected (Supplementary Table S2).

### 3.3 Generalization of known PCOS loci and GxE interactions

**FIGURE 2:**
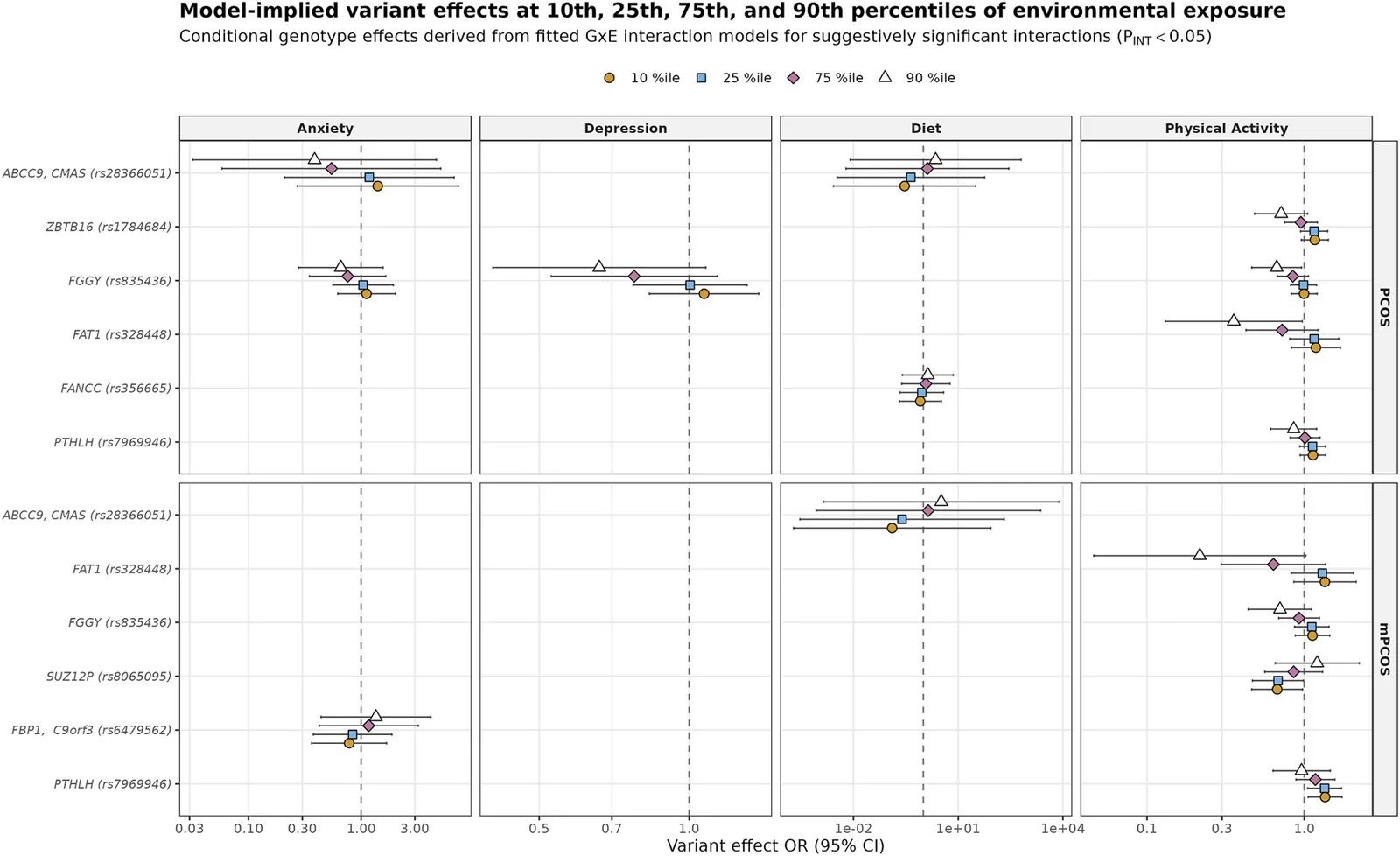
Model-implied genotype effects across environmental exposures. Forest plots display conditional genotype odds ratios and 95% confidence intervals evaluated at prespecified low and high values of the exposure (e.g., percentile-based values from the observed exposure distribution) derived from fitted G × E interaction models (*β_G_+eβ_G×E_*). Each row corresponds to a unique variant within each trait (PCOS, mPCOS), with columns representing environmental factors (anxiety, depression, diet, and physical activity). Effects are shown only in environments where suggestively significant (*P_INT_* < 0.05) interactions were identified. Confidence intervals are approximate due to unavailable covariance between main and interaction effects. Interaction statistics and their gene consequences are described in detail in Table 5 **.**

We used summary statistics from Hayes et al. study, conducted in individuals of European ancestry, and incorporated 31 independent SNPs associated with PCOS at suggestive genome-wide significance (Hayes et al., 2015) to evaluate these loci’s generalizability to reproductive aged Hispanic/Latinas. Hayes and colleagues identified two novel variants in/near *GATA4/NEIL2* locus and *FSHB/ARL14EP* locus (rs804279 and rs11031006, respectively) and replicated a previous association in Chinese populations in the *FANCC/C9orf3* locus (rs10993397). We were not able to generalize the effects at these loci in our population (Table 4). However, significant effects of SNPs in regions chr7q11.22 (at *STAG3L4* locus*)* and chr17q21.33 (at rs198536, intronic to *CACNA1G* antisense RNA 1) were seen with both PCOS and mPCOS at suggestive significance (p < 0.05). While in the *C9orf3/FANCC* locus no significant association was observed with PCOS, a significant positive interaction of diet with an intronic variant in this region was observed on PCOS with suggestive significance (rs356665, *β =* 0.03, *SE* = 0.01, p_INT_<0.05) (Table 5; Figure 2). Variants rs328448 (an intronic *FAT1* variant), and rs7969946 (140 kb 5ʹ of *PTHLH*) had marginally significant negative interactions with physical activity on PCOS and mPCOS (all p_INT_<0.05). Variant rs28366051 in chr12p12.1, 158 bp 5ʹ of *CMAS,* demonstrated a significant positive interaction effect with diet on both PCOS (*β =* 0.11, *SE* = 0.04, p_INT_ = 0.002) and mPCOS (*β =* 0.18, *SE* = 0.05, p_INT_ = 0.001) and a significant negative interaction with anxiety on PCOS (*β =* −0.09, *SE* = 0.04, p_INT_ = 0.033). While an intronic variant in *FGGY* (rs835436) showed no significant main effect on PCOS, it exhibited a significant negative interaction with anxiety symptoms (*β =* −0.04, *SE* = 0.01, p_INT_ = 0.01) and depression symptoms on PCOS (*β =* −0.03, *SE* = 0.01, p_INT_ = 0.02) at suggestive significance. Moreover, interactions with physical activity appeared to be nominally attenuated with this variant on mPCOS (*β =* −0.0003, *SE* = 0.0001, p_INT_ = 0.02) (Figure 2). A variant in intron two of *ZBTB16* (rs1784684) also displayed a significant negative interaction with physical activity on PCOS (*β =* −0.0004, *SE* = 0.0001, p_INT_ = 0.01). We also observed a nominally significant positive interaction between anxiety and a variant 26 kb 5ʹ of *SUZ12P* on mPCOS (*β =* 0.0004, *SE* = 0.0002, p_INT_ = 0.019). Lastly, variant rs6479562 located in chromosome nine open reading frame 118 and 27 kb upstream of *FBP1* exhibited a nominally significant positive interaction with depression symptoms on mPCOS (*β =* 0.04, *SE* = 0.02, p_INT_ = 0.04). In spite of these nominal associations, there were no significant GxE interactions that remained after correcting for multiple tests (p < 0.0004).

**TABLE 4:** Generalizing previous GWAS associations with PCOS and the metabolic subtype in HCHS/SOL (n = 1,698).

Top signal in discovery population				Top signal in our population at the locus													
Signal in Hayes et al., 2015				PCOS generalization							Metabolic subtype generalization						
Variant	Chr:Position	Pmeta	Nearest gene	Variant	Chr:Position	EA	NEA	EAF	OR (95%CI)	P-value	Variant	Chr:Position	EA	NEA	EAF	OR (95%CI)	P-value
rs12732606	chr1:8279450	4.33E-06	*LOC100129776, SLC45A1*	rs12732606	chr1:8279450	G	A	0.832	1.02 (0.82–1.27)	0.858	rs12732606	chr1:8279450	G	A	0.830	0.89 (0.67–1.18)	0.422
rs835436	chr1:59374380	8.04E-06	*LOC729467, FGGY, HOOK1*	rs835436	chr1:59374380	C	T	0.414	0.88 (0.75–1.02)	0.096	rs835436	chr1:59374380	C	T	0.420	0.94 (0.77–1.16)	0.579
rs2848118	chr2:88823670	1.12E-02	*FLJ40330, LOC100132330, IGKDEL*	rs2848118	chr2:88823670	C	G	0.102	0.88 (0.68–1.13)	0.312	rs2848118	chr2:88823670	C	G	0.102	0.85 (0.60–1.21)	0.371
rs7574059	chr2:123613428	2.54E-07	*LOC728241, LOC100131284*	rs4146018	chr2:123724441	T	C	0.633	0.91 (0.77–1.07)	0.264	rs11122996	chr2:123673536	T	A	0.705	1.17 (0.93–1.48)	0.169
rs10174494	chr2:240355845	8.04E-06	*OTOS, GPC1*	rs10174494	chr2:240355845	A	G	0.328	1.14 (0.96–1.35)	0.131	rs10174494	chr2:240355845	A	G	0.326	1.13 (0.90–1.41)	0.298
rs2881479	chr3:12325606	2.33E-06	*GSTM1L, PPARG PPARG, TSEN2*	rs2881479	chr3:12325606	T	A	0.110	1.09 (0.85–1.39)	0.518	rs2881479	chr3:12325606	T	A	0.109	1.06 (0.77–1.47)	0.705
rs36118024	chr3:14981336	1.03E-05	*FGD5, NR2C2, MRPS25*	rs36118024	chr3:14981336	G	C	0.308	1.06 (0.90–1.25)	0.481	rs36118024	chr3:14981336	G	C	0.309	1.10 (0.88–1.37)	0.389
rs9755867	chr3:86861115	6.97E-06	*CADM2, VGLL3*	rs9755867	chr3:86861115	T	C	0.686	0.95 (0.81–1.13)	0.582	rs9755867	chr3:86861115	T	C	0.686	0.94 (0.76–1.17)	0.588
rs3856928	chr3:186595782	1.15E-06	*DNAJB11, AHSG*	rs3856928	chr3:186595782	A	T	0.767	0.89 (0.74–1.06)	0.196	rs3856928	chr3:186595782	A	T	0.768	0.87 (0.68–1.1)	0.242
rs328448	chr4:186663848	2.02E-06	*MTNR1A, FAT, MRPS36P2*	rs328448	chr4:186663848	C	T	0.078	0.85 (0.63–1.14)	0.268	rs328448	chr4:186663848	C	T	0.079	0.85 (0.58–1.25)	0.413
rs154766	chr5:115754440	1.07E-02	*TICAM2, CDO1*	rs154766	chr5:115754440	G	T	0.176	1.20 (0.98–1.47)	0.077	rs154766	chr5:115754440	G	T	0.169	1.04 (0.79–1.37)	0.777
-	chr6:27790065	4.52E-06	​	-	chr6:27790065	A	AG	0.067	1.06 (0.78–1.44)	0.715	-	chr6:27790065	A	AG	0.070	1.31 (0.90–1.90)	0.154
rs7797476	chr7:67628747	2.99E-04	*STAG3L4, AUTS2*	**rs62457065**	**chr7:67614264**	**G**	**A**	**0.159**	**1.23 (1.00–1.52)**	**0.047**	**rs1109786**	**chr7:67594309**	**T**	**G**	**0.185**	**1.29 (1.01–1.66)**	**0.043**
rs804279	chr8:11766380	1.87E-08	*C8orf49, NEIL2*	rs804290	chr8:11759327	A	G	0.138	1.19 (0.96–1.47)	0.119	rs8191589	chr8:11776685	A	T	0.144	1.24 (0.95–1.63)	0.118
rs72650439	chr8:60665848	8.84E-06	*NA*	rs72650439	chr8:60665848	T	C	0.048	1.09 (0.77–1.55)	0.626	rs72650439	chr8:60665848	T	C	0.048	1.11 (0.69–1.77)	0.672
rs3996232	chr9:94137495	2.28E-06	*CRB2, DENND1A, LHX2*	rs3996232	chr9:94137495	A	C	0.308	1.04 (0.88–1.24)	0.630	rs3996232	chr9:94137495	A	C	0.311	1.24 (0.99–1.54)	0.063
rs10993397	chr9:94917489	3.07E-11	*FBP1, C9orf3, hCG_2003663*	rs10761387	chr9:95365062	C	G	0.589	0.92 (0.79–1.08)	0.330	rs10761387	chr9:95365062	C	G	0.587	0.86 (0.70–1.07)	0.170
rs10818866	chr9:123753010	4.58E-06	*CRB2, DENND1A, LHX2*	-	chr9:1,23818704	CA	C	0.057	0.89 (0.63–1.25)	0.488	rs10818889	chr9:123899478	G	A	0.061	1.14 (0.75–1.72)	0.542
rs11031006	chr11:30204981	8.48E-12	*KCNA4, FSHB*	rs12223987	chr11:30374324	T	C	0.123	0.87 (0.67–1.12)	0.272	rs540948	chr11:30213235	C	T	0.544	1.17 (0.95–1.44)	0.144
rs1784692	chr11:114078510	6.10E-07	*HTR3A, ZBTB16, NNMT*	rs3018331	chr11:114069747	A	C	0.171	1.04 (0.84–1.28)	0.724	rs1784692	chr11:114078510	C	T	0.171	0.90 (0.68–1.19)	0.467
rs28366051	chr12:22046065	8.95E-06	*ABCC9, CMAS, CMAS*	rs28366051	chr12:22046065	C	T	0.034	0.88 (0.57–1.36)	0.562	rs28366051	chr12:22046065	C	T	0.034	0.96 (0.54–1.70)	0.895
-	chr12:28567553	1.32E-07	​	rs12317339	chr12:28518389	G	T	0.486	0.90 (0.77–1.04)	0.155	rs4035202	chr12:28118015	G	A	0.543	1.20 (0.98–1.48)	0.079
rs10083228	chr13:39957995	3.49E-06	*COG6, LOC646982*	rs10083228	chr13:39957995	C	G	0.013	1.24 (0.62–2.48)	0.535	rs10083228	chr13:39957995	C	G	0.012	1.05 (0.41–2.73)	0.915
rs7337751	chr13:71321720	5.51E-06	*ATXN8OS, DACH1*	rs7337751	chr13:71321720	G	T	0.733	0.85 (0.72–1.02)	0.077	rs7337751	chr13:71321720	G	T	0.736	0.88 (0.70–1.10)	0.265
rs1760903	chr14:20384658	7.09E-06	*PARP2, TEP1, KLHL33*	rs1760903	chr14:20384658	A	G	0.602	0.94 (0.80–1.10)	0.430	rs1760903	chr14:20384658	A	G	0.601	0.88 (0.71–1.08)	0.225
rs76519333	chr15:34655432	2.82E-06	*NA*	rs16959273	chr15:34643056	A	G	0.096	1.08 (0.83–1.40)	0.568	rs141899334	chr15:34653512	C	T	0.094	0.87 (0.60–1.26)	0.475
rs12446759	chr16:81739398	3.02E-06	*CMIP, PLCG2*	rs12446759	chr16:81739398	A	G	0.526	0.97 (0.83–1.13)	0.718	rs12446759	chr16:81739398	A	G	0.527	0.98 (0.80–1.20)	0.850
rs11653259	chr17:30685799	8.80E-06	*LRRC37B2, SUZ12P*	rs8065095	chr17:30683616	C	T	0.160	1.06 (0.86–1.30)	0.590	rs8065095	chr17:30683616	C	T	0.156	0.87 (0.65–1.17)	0.350
rs198554	chr17:50581041	6.23E-06	*LOC253962, CACNA1G-AS1*	**rs198536**	**chr17:50559636**	**C**	**T**	**0.143**	**1.26 (1.01–1.57)**	**0.037**	**rs198536**	**chr17:50559636**	**C**	**T**	**0.139**	**1.34 (1.01–1.79)**	**0.044**
rs6133068	chr20:3861142	7.68E-04	*C20orf29, VISA, PANK2*	rs6133068	chr20:3861142	A	G	0.090	1.12 (0.86–1.46)	0.390	rs6133068	chr20:3861142	A	G	0.088	1.01 (0.70–1.46)	0.938
-	chr21:20881957	4.07E-06	​	-	chr21:20881957	A	AT	0.033	1.30 (0.85–1.99)	0.232	**-**	**chr21:20881957**	**A**	**AT**	**0.034**	**1.68 (1.02–2.78)**	**0.042**

Generalization in Hispanic/Latina reproductive aged women of genome-wide significant variants from Hayes et al., 2015 study of European population are shown here in Odds ratios and 95% Confidence intervals. None of the discovery single nucleotide polymorphisms (rows highlighted in dark gray) were generalized in our sample. Significance was evaluated at bonferroni corrected p-value threshold of 0.002 for 31 independent SNPs tested. And suggestive significance was evaluated at a p-value threshold of 0.05. Genetic associations were adjusted for age and first 5 genetic ancestry principal components for both phenotypes. We accounted for genetic relatedness and HCHS/SOL complex survey sampling in our regression models using SUGEN v8.9. Effects with best proxies of the variants in HCHS/SOL (within 500 kb of Hayes signal) are provided and those with suggestive significance have been bolded. Chromosome and position on GRCh38 genomic build, variant rsID (wherever available), effect allele, non-effect allele, effect allele freqeuncy, and the nearest gene to the variant have been listed in the table.

**TABLE 5:** Gene by Environment interactions with known PCOS loci in HCHS/SOL.

Outcome = PCOS															
​									Genetic main effect		E main effect		Interaction (GxE)		P_JOINT_
Environment	Variant	SNP ID	EA	NEF	EAF	N_INFO	N_CASE	Nearest Gene(s)	B(SE)	P_G_	B(SE)	P_E_*	B(SE)	P_INT_	​
Diet quality (AHEI-2010)	rs356665	chr9:95298349	C	T	0.45	1,698	437	*FANCC*	−1.25 (0.55)	0.023	−3.41E-02 (1.38E-02)	0.013	2.80E-02 (1.18E-02)	0.018	0.053
Diet quality (AHEI-2010)	rs28366051	chr12:22046065	C	T	0.03	1,698	437	*ABCC9, CMAS*	−5.55 (1.74)	0.001	−1.63E-02 (9.19E-03)	0.076	1.15E-01 (3.64E-02)	0.002	0.006
Total physical activity (MET-min/day)	rs835436	chr1:59374380	C	T	0.41	1,698	437	*FGGY*	−1.26E-3 (0.98)	0.990	2.49E-04 (1.15E-04)	0.030	−2.94E-04 (1.16E-04)	0.012	0.010
Total physical activity (MET-min/day)	rs328448	chr4:186663848	C	T	0.08	1,698	437	*FAT1*	0.17 (0.18)	0.351	7.76E-05 (7.71E-05)	0.315	−8.78E-04 (3.49E-04)	0.012	0.037
Total physical activity (MET-min/day)	rs1784684	chr11:114068817	G	A	0.32	1,698	437	*ZBTB16*	0.16 (0.1)	0.125	2.12E-04 (9.79E-05)	0.030	−3.61E04 (1.30E-04)	0.006	0.021
Total physical activity (MET-min/day)	rs7969946	chr12:28111814	A	G	0.49	1,698	437	*PTHLH, LOC100129646*	0.13 (0.1)	0.182	2.10E-04 (1.22E-04)	0.085	−2.06E-04 (1.02E-02)	0.044	0.126
Anxiety symptoms (STAI)	rs835436	chr1:59374380	C	T	0.41	1,698	437	*FGGY*	0.50 (0.26)	0.056	4.24E-02 (1.36E-02)	0.002	−3.52E-02 (1.38E-02)	0.011	0.010
Anxiety symptoms (STAI)	rs28366051	chr12:22046065	C	T	0.03	1,698	437	*ABCC9, CMAS, CMAS*	1.29 (0.71)	0.069	2.24E-02 (9.56E-03)	0.019	−8.62E-02 (4.04E-02)	0.033	0.088
Depression symptoms (CESD10)	rs835436	chr1:59374380	C	T	0.41	1,698	437	*FGGY*	0.10 (0.13)	0.430	3.76E-02 (1.33E-02)	0.005	−3.22E-02 (1.35E-02)	0.017	0.015

Outcome = mPCOS															
​	​	​	​	​	​	​	​	​	Genetic main effect		​	E main effect	Interaction (GxE)		P_JOINT_
Environment	Variant	SNP ID	EA	NEF	EAF	N_INFO	N_CASE	Nearest Gene(s)	B(SE)	P_G_	B(SE)	P_E_*	B(SE)	P_INT_	
Diet quality (AHEI-2010)	rs28366051	chr12:22046065	C	T	0.03	1,478	217	*ABCC9, CMAS*	−8.89 (2.66)	0.001	−1.06E-02 (1.20E-02)	0.377	1.82E-01 (5.30E-02)	0.001	0.002
Total physical activity (MET-min/day)	rs835436	chr1:59374380	C	T	0.42	1,478	217	*FGGY*	0.12 (0.13)	0.346	3.78E-04 (1.43E-04)	0.008	−3.49E-04 (1.46E-04)	0.017	0.053
Total physical activity (MET-min/day)	rs328448	chr4:186663848	C	T	0.08	1,478	217	*FAT1*	0.30 (0.23)	0.194	1.76E-04 (9.46E-05)	0.063	−1.34E-03 (5.43E-04)	0.014	0.049
Total physical activity (MET-min/day)	rs7969946	chr12:28111814	A	G	0.50	1,478	217	*PTHLH, LOC100129646*	0.31 (0.13)	0.015	3.43E-04 (1.50E-04)	0.023	−2.51E-04 (1.26E-04)	0.047	0.038
Anxiety symptoms (STAI)	rs8065095	chr17:30683616	C	T	0.16	1,478	217	*LOC105371723, SUZ12P*	−0.40 (0.19)	0.037	−4.69E-05 (1.20E-04)	0.695	4.29E-04 (1.82E-04)	0.019	0.043
Depression symptoms (CESD10)	rs6479562	chr9:94666878	G	A	0.67	1,478	217	*FBP1, C9orf3*	−0.65 (0.34)	0.055	−2.81E-02 (2.74E-02)	0.305	3.66E-02 (1.78E-02)	0.039	0.119

Nominally significant gene-by-environment interactions with GWAS significant SNPs from Hayes et al. and four environmental factors (Diet, Physical Activity, Anxiety, and Depression) are presented with corresponding effect sizes and p-values. Analyses were conducted in SUGEN v8.9, which accounts for the complex survey design and sample relatedness of HCHS/SOL. Regression models were adjusted for age and the first five genetic ancestry principal components.

We report the Genetic Main Effect [Beta (G)], Environment Main Effect [Beta (E)], the Interaction Effect [Beta (G × E)], and their respective Standard Errors (SE) calculated from the variance-covariance estimates. Statistical significance is provided for both the Joint 2-df Test (P-Joint), which evaluates the combined genetic and interaction signal, and the Interaction 1-df Test (P-Interaction), which evaluates environmental modification of the genetic effect. Significance was evaluated at bonferroni corrected p-value threshold of 0.0004 for 31 independent SNPs tested across 4 lifestyle factors. Suggestive significance was evaluated at a p-value threshold of 0.05. Corresponding rsIDs and nearest genes are provided for variants previously associated with PCOS.

*P-value for environmental main effect was calculated *post hoc* by calulcating the p-value of the wald statistic which was derived using the beta coefficient for the environment main effect (BETA_envi) and its variance estimate (COV_envi_envi) outputted by SUGEN. We used the formulae wald_statistic=(beta_e^2)/variance_e & pvalue_e_wald = pchisq (wald_statistic, df = 1, lower.tail = FALSE) in R.

## 4 Discussion

PCOS is a disease that has varying symptomology across individuals. The manifestation of PCOS can include any of the following: metabolic dysregulation (Meyer et al., 2020; Carmina et al., 2006; Wijeyaratne et al., 2006), insulin resistance (Farrell and Antoni, 2010; Ovalle and Azziz, 2002; Pugeat and Ducluzeau, 1999), irregular menses (Rosenfield, 2015), infertility (van der Spuy and Dyer, 2004; Legro and Strauss, 2002), and mental health issues (Hollinrake et al., 2007; Conte et al., 2015; Açmaz et al., 2013; Himelein and Thatcher, 2006). In fact, latest research has officially renamed PCOS to polyendocrine metabolic ovarian syndrome (PMOS) given the high metabolic burden observed in patients in combination with inconsistent presentation of ovarian cysts and a high prevalence of insulin resistance (Teede et al., 2026). With predisposition to higher waist-to-hip ratio, lipid dysregulation, and insulin resistance (Sam et al., 2015; Kazemi et al., 2022; Abruzzese et al., 2023) compared to their non-Hispanic White counterparts, Hispanic/Latinas with PCOS (Meyer et al., 2020; Mortada and Williams, 2015) are at a higher risk for MetS and thereby CVD, irrespective of their weight (Mortada and Williams, 2015), and this makes the population an important one to study for PCOS/PMOS. GWAS have revealed genomic regions that may play a role in the etiopathology of PCOS, including regions surrounding *THADA, DENND1A1* (Hiam et al., 2019)*, SHBG,* and *ERBB4* (Zhang Y. et al., 2020)*.* Yet, the gene by environment interactions that could shape these mechanisms and affect downstream CVD outcomes in this population have not been systematically explored. Evidence for the differential susceptibility model suggests that certain genotypes do not merely confer risk, but rather serve as ‘environmental sensors’ (Belsky, 2013; Dalle Molle et al., 2017). This results in a crossover interaction where the same variant associated with negative outcomes under chronic stress also predicts optimal functioning in low-stress environments (Belsky, 2013; Dalle Molle et al., 2017). Previous research indicates that BMI genetic risk scores interact with diet quality, US nativity (defined as born in 50 US states or District of Columbia) and acculturation (Fernández-Rhodes et al., 2023), with significantly larger GxE interactions observed in females. The San Antonio Family Heart Study (Mitchell et al., 1996) and the Portuguese Healthy Family Study (Santos et al., 2014) also highlight the importance of studying GxE interactions (e.g., gene by energy expenditure) in this population. Present study operationalized the metabolic subtype of PCOS in the reproductive aged Hispanic/Latinas, generalized prior genetic associations to this population with PCOS such as at the *STAG3L4* locus and the *CACNA1G* locus, and observed nominal GxE interaction effects on both PCOS and mPCOS.

We detected nominally significant interactions between diet and a variant (rs356665) in *FANCC* on both PCOS and mPCOS and diet and a variant near *CMAS* on PCOS (Figure 2). The Fanconi anemia complementation group C affects chromosomal stability and is linked to post-replication DNA repair and cell cycle checkpoint function (Gordon and Buchwald, 2024). Our variant, rs356665 in *FANCC* has been previously found to be associated with lung adenocarcinoma (Yang et al., 2016). In addition to PCOS, variants in this gene have been previously linked to breast cancer risk and loss of function studies elucidate their role in germ cell proliferation (Danbaki et al., 2025; Dörk et al., 2019). Our results suggest that individuals with the rs356665-C allele may have a reduced capacity to mitigate diet-induced cellular stress, thereby increasing susceptibility to the endocrine and metabolic characteristics of PCOS. Intronic variants in both *FGGY* (rs835436) and *FAT1* (rs328448) exhibited a suggestively significant negative interaction with physical activity on both PCOS and mPCOS in Hispanic/Latinas of reproductive age. GWAS have shown significant effects of variants in *FGGY,* a carbohydrate kinase domain containing gene, on sporadic amyotrophic lateral sclerosis (Dunckley et al., 2007). Variants in this gene were also linked to cellular atrophy-related pathways that affect muscle wasting (Smith et al., 2021) and colorectal cancer (Liu et al., 2025). The synergistic interaction between physical activity and rs835436-C in our study, albeit weak, suggests a potential protective effect of exercise on PCOS risk. Given that this interaction effect was noted for both PCOS and mPCOS, it could signify that the C-allele may enhance the physiological response to physical exertion. In these individuals, exercise may more effectively counteract the insulin resistance or metabolic dysfunction central to PCOS pathogenesis, potentially through improved muscle-mediated glucose uptake or proteostasis. Conversely, the suggestively significant negative main effect of the rs835436-C allele could have interacted with anxiety symptoms to reduce PCOS risk, potentially signifying that psychological stress may provide a potential “buffering” effect of this variant’s effect on PCOS in this population. Longitudinal studies of mental health factors and physical activity exploring their genetic interaction effects on PCOS could illuminate these GxE interations further. Further studies with larger sample sizes would be required to observe robust GxE interactions in this population.

A suggestively significant interaction effect of physical activity and a rs328448-C in the *FAT1* locus on both PCOS and mPCOS was also observed, however it was not significant after correcting for multiple tests. *FAT1,* FAT Atypical Cadherin 1, is a protein coding gene, which affects MAPK/ERK signaling pathways and cell proliferation with a potential role in tumor development (Peng et al., 2021). The nominal negative interaction observed between rs328448-C and physical activity suggests that exercise likely acts as a critical external regulator that mitigates the pro-proliferative and inflammatory signals associated with the MAPK pathway, thereby reducing the manifestation of PCOS and its metabolic subtype. The antagonistic nature of the interaction between physical activity and variants in *ZBTB16* (rs1784684-G) and *PTHLH* (rs7969946-A) further exemplifies the protective nature of exercise; likely reflecting the modulation of adipogenesis and insulin sensitivity (Wei et al., 2013; Krupková et al., 2018) and parathyroid hormone-related signaling pathways (Chang et al., 2017). However, further studies in larger Hispanic/Latina samples should validate these associations.

Anxiety and a variant near *SUZ12P* gene moderated the risk of mPCOS with suggestive significance, specifically, we observed that the potential protective effect of rs8065095 in individuals carrying the C allele in our population was negated by the presence of more anxiety symptoms. While mutations in the *SUZ12* gene have been previously linked to myeloproliferative neoplasms (Brecqueville et al., 2011), and other variants in the gene linked to a range of anthropometric phenotypes (Genecards.org, 2026; Stelzer et al., 2016), its role is largely unknown in the context of PCOS. A similar antagonistic interaction between rs6479562-G allele and depression symptoms was seen to exacerbate the risk of mPCOS. Variant rs6479562-G is located near *FBP1*, fructose-1,6-bisphosphatase gene, which has functions in gluconeogenesis (Zhang et al., 2024; Park et al., 2020) and tumor growth (Wang et al., 2025; Wang et al., 2022). The role of these genes and their interaction with mental health factors such as anxiety and depression should be further validated in large studies of Hispanic/Latinas with PCOS, given the higher prevalence of anxiety and depressive disorders in this population.

Our study has multiple strengths. For example, we leveraged the large, community-based HCHS/SOL cohort, enabling assessment of cardiometabolic, anthropometric, and environmental factors. We generalized known PCOS loci from predominantly European ancestry GWAS to Hispanic/Latina females and identified several biologically meaningful GxE interactions. However, we also have some limitations, including limited sample size for PCOS and mPCOS, which reduced power to detect additional associations. Our GxE interactions were underpowered to detect interactions and therefore, here, we summarize nominally significant interactions (p < 0.05), but note that none remain significant after correcting for multiple tests. Moreover, while we discussed the biological functions of our top variants and their nearest genomic regions, it should be noted that causal variants in LD can sometimes be up to 2 Mb away from the associated SNP (Brodie et al., 2016). Therefore, further analyses such as finemapping may be required to ascertain the causal variant in these loci in Hispanic/Latina females. We also recognize that reliance on self-reported PCOS may have introduced misclassification due to underdiagnosis and cultural or healthcare access barriers (Meyer et al., 2020; Rao et al., 2024; Engmann et al., 2017), and underestimate effects (Kim et al., 2023; Lin et al., 2018). Furthermore, we applied a European ancestry-derived PRS to Hispanic/Latinas and observed that there was poor transferability of the PRS in our population. Differing LD structures, higher admixture in Hispanic/Latinas, as well as genomic complexity and diversity in this population make the transferability of a European ancestry-derived PRS challenging. Efforts to conduct large-scale GWAS of PCOS in multi-ancestry populations including Hispanic/Latinas should be carried out. Future studies should validate our findings in larger, clinically confirmed cohorts to improve genetic risk prediction and mechanistic understanding in Hispanic/Latinas.

With recent growth in the US Hispanic/Latino population, it accounted for more than half of US population growth between 2022 and 2023 (U.S. Census Bureau, 2024). And even though this growth in Hispanic/Latinos is slower than previous years’ (1.8% between 2022 and 2023% vs. 2.0% between 2012 and 2013), it is still faster than the nation’s non-Hispanic population growth (0.2% between 2022 and 2023) (U.S. Census Bureau, 2024). Thus, it is of great clinical importance to identify high-risk individuals for cardiometabolic disease in this population, which can be exacerbated in PCOS patients (Engmann et al., 2017). Studying PCOS and cardiometabolic health in this population is crucial, and translation of genetic associations into clinically usable gene targets could enable clinicians to identify high-risk individuals. Present research could potentially guide future research and inform gene-environment pathways that could improve lifestyle management in PCOS patients.

## Data Availability

The original contributions presented in the study are included in the article/Supplementary Material, further inquiries can be directed to the corresponding author.
