# Supplementary material for: Gene by environment interaction effects on the metabolic subtype of Polycystic Ovary Syndrome in Hispanic Community Health Study/Study of Latinos
Source: Front Genet. 2026 Aug 28;17:1801152. doi: 10.3389/fgene.2026.1801152 (PMC13564261; doi:10.3389/fgene.2026.1801152)
Supplement: Supplementary file 1 [file Supplementaryfile1.docx]

Supplemental Materials

***Supplemental Figure 1 Polygenic risk score prediction performance***

Area under covariate-adjusted receiver operating characteristics curve (AROC AUC) has been shown here for a PCOS PRS predicting PCOS (**Supp Figure 1A**) and predicting mPCOS (**Supp** **Figure 1B**)**.** Both models were adjusted for age and first 5 principal components of genetic ancestry.

| **Supplemental Table 1. Quality control of previous GWAS variants in HCHS/SOL study** | | | | | | |  |  |  |  |  |  |  |  |  |  |
| --- | --- | --- | --- | --- | --- | --- | --- | --- | --- | --- | --- | --- | --- | --- | --- | --- |
|  | Hayes et al. 2015 | | | | | |  |  |  |  |  |  |  |  |  |  |
| Filter | SNPs kept | | SNPs lost | | | |  |  |  |  |  |  |  |  |  |  |
| Variants attempted | 340 | |  | | | |  |  |  |  |  |  |  |  |  |  |
| Not in HCHS/SOL data | 340 | | 0 | | | |  |  |  |  |  |  |  |  |  |  |
| MAF>0.01 | 337 | | 1 | | | |  |  |  |  |  |  |  |  |  |  |
| call rate < 90% | 337 | | 0 | | | |  |  |  |  |  |  |  |  |  |  |
| MAC>5 | 337 | | 0 | | | |  |  |  |  |  |  |  |  |  |  |
| HWE p-value < 1e-5 | 329 | | 8 | | | |  |  |  |  |  |  |  |  |  |  |
| Total | 329 | | 11 | | | |  |  |  |  |  |  |  |  |  |  |
| Abbreviations: GWAS, Genome-wide associations study; MAF, Minor allele frequency; MAC, Minor allele count; SNP, single nucleotide polymorphism | | | | | | |  |  |  |  |  |  |  |  |  |  |
|  |  |  |  |  |  |  |  | | | | | |  |  |  |  |
| **Supplemental Table 2. Regression results of PCOS PRS by environment interactions** | | | | | | | | | | | | | | | | |
|  | | Outcome = PCOS | | | | | | | | Outcome = mPCOS | | | | | | |
|  | | Effect of factor | | | Effect of PRS | | | Interaction Effect | | Effect of factor | | Effect of PRS | | | Interaction Effect | |
| **Environmental factors** | | OR (95% CI) | | *p* value | OR (95% CI) | *p* value | | OR (95% CI) | *p* value | OR (95% CI) | *p* value | OR (95% CI) | | *p* value | OR (95% CI) | *p* value |
| Diet quality (AHEI) | | 0.98 (0.96-1.00) | | 0.102 | 0.76 (0.33-1.73) | 0.515 | | 1.01 (0.99-1.02) | 0.564 | 0.98 (0.95-1.01) | 0.128 | 0.89 (0.29-2.76) | | 0.844 | 1.00 (0.98-1.03) | 0.845 |
| Total physical activity (MET-min/day)) | | 1.00 (1.00-1.00) | | 0.910 | 0.95 (0.83-1.09) | 0.499 | | 1.00 (1.00-1.00) | 0.587 | 1.00 (1.00-1.00) | 0.462 | 1.02 (0.85-1.22) | | 0.844 | 1.00 (1.00-1.00) | 0.903 |
| Anxiety Symptoms (STAI10) | | 1.01 (1.00-1.03) | | 0.128 | 0.82 (0.58-1.17) | 0.276 | | 1.01 (0.99-1.03) | 0.321 | 1.02 (0.99-1.04) | 0.145 | 0.97 (0.61-1.56) | | 0.911 | 1.00 (0.98-1.03) | 0.859 |
| Depressive Symptoms (CESD10) | | 1.01 (0.99-1.03) | | 0.174 | 0.90 (0.75-1.08) | 0.262 | | 1.01 (0.99-1.03) | 0.313 | 1.01 (0.98-1.03) | 0.478 | 1.00 (0.79-1.28) | | 0.973 | 1.00 (0.98-1.02) | 0.946 |
| **Cardiometabolic factors** | |  | |  |  |  | |  |  |  |  |  | |  |  |  |
| BMI, kg/m^2^ | | 1.02 (1.00-1.04) | | 0.015 | 0.82 (0.49-1.36) | 0.439 | | 1.01 (0.99-1.02) | 0.485 | -* | <0.0001 | 0.78 (0.37-1.65) | | 0.511 | 1.01 (0.99-1.03) | 0.435 |
| Waist to hip ratio at V2 | | 22.35 (4.07-124.27) | | <0.001 | 0.32 (0.07-1.50) | 0.149 | | 3.45 (0.62-19.37) | 0.158 | -* | <0.0001 | 0.25 (0.03-2.25) | | 0.22 | 4.43 (0.42-47.38) | 0.217 |
| SBP, mmHg | | 1.00 (0.99-1.01) | | 0.821 | 1.06 (0.47-2.40) | 0.887 | | 1.00 (0.99-1.01) | 0.835 | 1.00 (0.99-1.01) | 0.428 | 0.37 (0.12-1.09) | | 0.073 | 1.01 (1.00-1.02) | 0.069 |
| DBP, mmHg | | 1.01 (0.99-1.03) | | 0.174 | 0.90 (0.75-1.08) | 0.262 | | 1.01 (0.99-1.03) | 0.313 | 1.01 (0.98-1.03) | 0.478 | 1.00 (0.79-1.28) | | 0.973 | 1.00 (0.98-1.02) | 0.946 |
| Fasting Glucose, mg/dL | | 1.00 (1.00-1.01) | | 0.187 | 0.87 (0.62-1.24) | 0.438 | | 1.00 (1.00-1.00) | 0.571 | -* | <0.0001 | 0.98 (0.66-1.44) | | 0.904 | 1.00 (1.00-1.00) | 0.927 |
| Fasting Insulin, mg/dL | | 1.02 (1.01-1.02) | | 0.0013 | 0.95 (0.79-1.15) | 0.589 | | 1.00 (0.99-1.01) | 0.902 | -* | <0.0001 | 1.11 (0.82-1.48) | | 0.507 | 0.99 (0.98-1.01) | 0.393 |
| HOMA-IR | | 1.05 (1.02-1.08) | | <0.0001 | 0.95 (0.80-1.13) | 0.559 | | 1.00 (0.97-1.03) | 0.923 | 1.19 (1.14-1.24) | <0.0001 | 1.06 (0.82-1.37) | | 0.666 | 0.99 (0.95-1.02) | 0.457 |
| Triglycerides, mg/dL | | 1.00 (1.00-1.00) | | 0.030 | 0.92 (0.75-1.13) | 0.445 | | 1.00 (1.00-1.00) | 0.654 | 1.00 (1.00-1.01) | <0.0001 | 1.00 (0.76-1.32) | | 0.989 | 1.00 (1.00-1.00) | 0.994 |
| HDL, mg/dL | | 1.00 (0.99-1.00) | | 0.465 | 0.94 (0.61-1.45) | 0.788 | | 1.00 (0.99-1.01) | 0.931 | 0.96 (0.95-0.97) | <0.0001 | 0.74 (0.38-1.43) | | 0.367 | 1.01 (0.99-1.02) | 0.391 |
| LDL, mg/dL | | 1.00 (1.00-1.00) | | 0.684 | 0.99 (0.65-1.51) | 0.959 | | 1.00 (1.00-1.00) | 0.866 | 1.00 (1.00-1.01) | 0.129 | 1.19 (0.67-2.12) | | 0.546 | 1.00 (0.99-1.00) | 0.516 |
| Diabetes | | 1.72 (1.28-2.31) | | <0.0001 | 0.96 (0.84-1.09) | 0.525 | | 1.05 (0.78-1.40) | 0.762 | 3.42 (2.41-4.85) | <0.0001 | 0.98 (0.82-1.18) | | 0.826 | 1.04 (0.74-1.47) | 0.815 |
| Metabolic Syndrome | | 1.32 (1.02-1.70) | | 0.035 | 0.94 (0.82-1.08) | 0.388 | | 1.08 (0.84-1.39) | 0.556 | 3.34 (2.43-4.60) | <0.0001 | 1.01 (0.82-1.24) | | 0.960 | 0.96 (0.70-1.31) | 0.782 |
| Odds ratios and 95% confidence intervals of interactions between a Polygenic risk score (PRS) for PCOS derived from Day et al. 2018 summary statistics and study characteristics have been presented here. PRS by environment effects (diet, physical activity, anxiety, depression), by quantitative laboratory measures, and two categorical cardiometabolic outcomes were measured. Main effect estimates for the environmental factor, for the PRS, and the effect of the interaction between the two are summarized here. We did not detect any significant interactions with the PRS across any environmental factor. *Effect estimates for continuous lab measurements that were used to derive mPCOS were omitted from the table due to confounding. | | | | | | | | | | | | | | | | |
